# State-Dependent Temporal Dynamics of Short-Latency Afferent Inhibition Across Current Direction and Pulse Duration Using Controllable Pulse-Parameter TMS

**DOI:** 10.3390/brainsci16080800

**Published:** 2026-07-29

**Authors:** Kylee R. Graham, Sean K. Meehan

**Affiliations:** Department of Kinesiology and Health Sciences, University of Waterloo, 200 University Ave. W, Waterloo, ON N2L 3G1, Canada; krgraham@uwaterloo.ca

**Keywords:** sensorimotor integration, short-latency afferent inhibition, transcranial magnetic stimulation, controllable pulse parameter TMS, motor cortex, I-waves, intracortical circuits, muscle activation, cortical excitability, motor control

## Abstract

**Highlights:**

**What are the main findings?**

- Voluntary contraction shifted peak SAI to later interstimulus intervals and broadened the inhibitory window, independent of current direction and pulse duration.
- SAI magnitude increased during contraction for AP stimulation, whereas PA stimulation remained unchanged, suggesting current-direction-dependent modulation of inhibition magnitude.

**What are the implications of the main findings?**

- SAI may reflect multiple afferent-sensitive components with distinct temporal and functional properties rather than a single fixed response.
- Full temporal profiling of SAI, including timing, width and magnitude, provides greater physiological insight than single-ISI measures for studying somatosensory influence on motor output.

**Abstract:**

Background: Short-latency afferent inhibition (SAI) is widely used as a non-invasive measure of how somatosensory input modulates TMS-evoked motor output. However, SAI is modulated by motor state and stimulation parameters, suggesting that SAI may depend on the neural elements engaged by the stimulation protocol. This study examined how muscle activation state, transcranial magnetic stimulation (TMS) current direction, and pulse duration influence the temporal dynamics of SAI. Methods: Eighteen healthy adults completed two sessions in which SAI was assessed at rest and during a 10% maximum voluntary contraction of the first dorsal interosseous muscle. Within each session, controllable pulse-parameter TMS was used to profile SAI across interstimulus intervals (10–36 ms), current directions (posterior–anterior [PA], anterior–posterior [AP]), and pulse durations (30, 70, 120 µs) in a factorial design. Gaussian functions were used to estimate the peak magnitude, timing, and temporal width of inhibition in each condition. Effects were analyzed using linear mixed-effects models. Results: Voluntary contraction shifted peak inhibition to later ISIs and broadened the inhibitory window. AP stimulation yielded a broader inhibitory window than PA stimulation, whereas the peak magnitude of inhibition was increased during contraction for AP- but not PA-evoked responses. Conclusions: These findings indicate that motor state and stimulation parameters shape different features of the SAI temporal profile. Voluntary contraction altered the timing and width of inhibition, whereas current direction influenced the width and modulated the effect of muscle state on inhibition magnitude. SAI measured only at rest may not fully capture the state dependence of early somatosensory influences on motor cortical excitability; incorporating measurements across motor states and time courses may provide a more complete characterization.

## 1. Introduction

Somatosensory afference provides continuous information about body state and the immediate physical environment that can influence the excitability of motor cortical circuits involved in generating motor output. One widely used non-invasive method for probing somatosensory influence on motor cortical excitability is afferent inhibition [1,2]. In transcranial magnetic stimulation (TMS) studies, afferent inhibition is quantified as the suppression of the motor evoked potential (MEP) amplitude elicited by stimulation of the motor cortex when the TMS pulse is preceded by peripheral nerve stimulation [1,3,4]. Short-latency afferent inhibition (SAI) provides a neurophysiological index of the early influence of peripheral somatosensory input on corticospinal excitability [2].

Mechanistically, SAI is cortical in origin [3]. It is commonly attributed to a pathway linking peripheral afferents to M1 via thalamocortical projections to the primary somatosensory cortex (S1), which in turn modulates intracortical circuits within M1 [1,5]. The timing of maximal SAI, typically observed at interstimulus intervals (ISIs) of approximately 18–24 ms following median nerve stimulation [3,6,7], aligns closely with the arrival of afferent volleys in S1, as indexed by the N20 component of the somatosensory evoked potential [8]. This temporal correspondence, together with evidence linking SAI magnitude to the strength of afferent projections to the motor cortex, has supported the view that SAI reflects an early somatosensory influence on motor cortical excitability.

Despite this canonical framework, growing evidence suggests that the relationship between peripheral afferent input and the modulation of motor cortical excitability, as indexed by SAI, is not invariant. During the peak window of inhibition, SAI is reduced in the agonist motor cortical representation just before ballistic movement onset [9,10,11], but can be enhanced in adjacent motor representations not directly involved in the movement [10,12,13]. SAI is also modulated by attentional focus and verbal working memory load [11,14], indicating that the expression of afferent inhibition depends on the motor and cognitive state in which somatosensory afference is processed.

Anatomical and neurophysiological evidence also suggests that early afferent inhibition is not a unitary phenomenon. While SAI magnitude and N20 somatosensory evoked potential amplitude are highly correlated, SAI can persist in the absence of the N20 following focal lesions to the first-order somatosensory thalamic relay nuclei [15] whereas it can be reduced by lesions to the paramedian thalamic region that preserve the N20 [16]. These observations suggest that SAI reflects the net influence of somatosensory input on motor cortical output through multiple contributing pathways.

Studies that manipulate the direction of induced TMS current and, more recently, the duration of the TMS stimulus, further support the view that SAI reflects a composite response shaped by the afferent volley and the cortical elements recruited by TMS. Most studies have investigated SAI using posterior–anterior (PA)-induced current. However, anterior–posterior (AP)-induced current appears to recruit a different combination of excitatory inputs to the upper-limb motor cortical output neuron. Accordingly, the effect of the peripheral conditioning stimulus on the MEPs evoked by PA- or AP-induced current may reflect different components of early somatosensory modulation of motor cortical output. This possibility is supported by evidence that, at rest, the same peripheral nerve stimulus suppresses excitatory PA-evoked responses more strongly than AP-evoked responses [6]. Dissociations between PA and AP SAI have also been reported across motor state [6], task demands [17,18], and neurological injury [18]. The introduction of controllable-pulse-parameter TMS (cTMS) [19] has provided additional flexibility in manipulating pulse waveform characteristics, including pulse duration, to bias the recruitment of different neuronal elements [20,21]. For example, even within AP-induced current, SAI assessed with shorter (30 µs) pulses is differentially influenced by anodal cerebellar transcranial direct current stimulation compared to SAI assessed using longer (120 µs) duration pulses [21].

Together, these findings support a more nuanced view of SAI as a context-sensitive measure of early afferent modulation of motor cortical output. Current understanding remains limited by methodological constraints in the existing literature, as most studies have only employed PA-induced current with the fixed pulse characteristics of conventional stimulators [22,23], assessed SAI at rest and examined only one or two ISIs. Consequently, it remains unclear how the timing, magnitude and duration of SAI are shaped by the interaction between somatosensory afferent input, the cortical substrates acted on by the induced current and behavioral state.

The present study characterized the temporal profile of SAI and its modulation by muscle state, cTMS current direction, and pulse duration. Based on past work using a conventional stimulator [6], SAI was assessed across ISIs ranging from 10 to 36 ms in six stimulation conditions defined by the combination of current direction (PA, AP) and the duration of the positive phase of the TMS stimulus (30, 70, and 120 µs). SAI was measured both at rest and during a low-level voluntary contraction (10% maximum voluntary contraction) of the first dorsal interosseous muscle. Gaussian modelling of the SAI time course was used to quantify the timing of peak inhibition (μ), magnitude of inhibition (A), and the temporal width of the inhibitory window (full width at half maximum (FWHM)). This approach allowed us to move beyond single-ISI estimates and determine how stimulation parameters and motor state shape both the strength and temporal expression of afferent inhibition.

We hypothesized that muscle contraction would alter the temporal dynamics of SAI, including the timing, magnitude, and width of the inhibitory profile. Furthermore, we predicted that these effects would differ between PA- and AP-induced current, consistent with evidence that these current directions preferentially recruit partly distinct cortical inputs converging on the corticospinal neuron. Specifically, we expected AP stimulation to show greater modulation by muscle state, given prior evidence that AP-sensitive neuronal substrates are more variable and context-dependent. Finally, we hypothesized that pulse duration would interact with current direction to influence SAI dynamics, reflecting pulse-duration-dependent differences in neural elements recruited by the TMS pulse. By combining systematic manipulation of stimulation parameters with modelling of the full SAI time course, this study aimed to clarify how early somatosensory modulation of motor cortical output is shaped by the cortical circuitry preferentially engaged by different TMS configurations and by motor state.

## 2. Materials and Methods

### 2.1. Participants

Eighteen healthy adults (13 female, 22 ± 3 years old) with no history of neurological disease or contraindications to TMS completed two experimental sessions separated by at least forty-eight hours. Seventeen of the participants self-reported right-hand dominance. Participants were screened for contraindications to TMS using a modified screening questionnaire that included questions about health history and medications [24]. Participants were excluded if they reported a neurological injury (other than concussion), psychiatric or neurological disorder, were currently being treated for depression or were taking prescription medications that could influence the central nervous system. Participants were also screened for any implanted medical devices that could be adversely affected by the magnetic fields generated by the TMS stimulator. All participants reported obtaining at least six hours of sleep the previous night. All participants provided written informed consent, and this study was approved by the University of Waterloo’s Clinical Research Ethics Board (ORE #44090).

### 2.2. Experimental Design

Participants completed two sessions separated by at least forty-eight hours. In both sessions, SAI was quantified using MEPs from the right first dorsal interosseous muscle (FDI) elicited by cTMS over the contralateral motor cortex. In each session, SAI was assessed separately for cTMS stimuli by delivering TMS pulses with an initial positive phase of 30 µs, 70 µs or 120 µs that induced either a posterior–anterior (PA_30_, PA_70_ or PA_120_) or anterior–posterior (AP_30_, AP_70_ or AP_120_) current in the underlying tissue. SAI was assessed for all six current direction-pulse duration combinations within each session, with one 156-trial block completed for each combination. Within each 156-trial block, there were 12 unconditioned stimuli and 144 stimuli in which peripheral nerve stimulation preceded TMS by one of 12 possible intervals. Therefore, each session included six stimulation blocks, for a total of 936 trials.

The only difference between sessions was the state of the FDI. In one session, SAI was assessed with the FDI at rest. In the other session, SAI was assessed while participants maintained a slight tonic contraction (10% of maximum voluntary contraction). Session order and the order of the six current direction-pulse duration blocks were randomized across participants. The trial structure within each block, including ISI order, unconditioned trials, intertrial interval, and rest breaks, is described in Section 2.4.

### 2.3. Controllable Pulse Parameter Transcranial Magnetic Stimulation (cTMS)

MEPs elicited by cTMS were recorded using LabChart 8 software, a Quad BioAmp and PowerLab 4/35 acquisition system (AD Instruments, Colorado Springs, CO, USA). Surface electromyography electrodes (Ag-AgCl) were placed over the FDI using a tendon-belly montage. Electromyographic recording was triggered using a 5 V TTL pulse with an epoch of −0.3 to 0.5 s. Data were amplified (×1000), digitized (×40,000 Hz) and filtered (bandpass filtered 5–1000 Hz, notch filter −60 Hz). Data were subsequently down-sampled to 5000 Hz during offline analysis. MEP amplitude was quantified on a trial-by-trial basis from the unrectified EMG signal as the peak-to-peak voltage difference between the maximum and minimum deflections within a fixed 20–50 ms window following TMS pulse onset. This window was selected to capture the expected corticomotor response from the first dorsal interosseous muscle while excluding the immediate stimulus artifact.

TMS was delivered using an Elevate cTMS stimulator (Rogue Research, Montreal, QC, Canada). The current direction was controlled using two 70 mm, medium-inductance (20 µH) figure-8 coils (Rogue Research, Montreal, QC, Canada). The physical geometry of the coils was identical; however, the manufacturer reversed the wiring of one coil so that the initial positive phase of the electric field induced an AP current in the underlying neural tissue. Pulse duration (e.g., 30, 70 or 120 µs) refers to the duration of the dominant initial positive phase of the TMS pulse and was controlled through the stimulator’s onboard control software. For all current direction-pulse duration combinations, the M-ratio was set to 0.2. The M-ratio represents the ratio of capacitor voltages driving the positive and negative phases of the TMS pulse. An M-ratio of one is a balanced biphasic stimulus where the strength of the positive and negative electric fields is equivalent. Decreasing the M-ratio reduces the magnitude of the biphasic stimulus’s negative (second) phase, introducing an imbalanced biphasic stimulus in which the initial positive field phase is responsible for the evoked response.

The FDI motor cortical hotspot was defined as the scalp position that elicited the largest and most consistent MEP in response to PA_70_ stimulation, with the coil held ~45° to the midline. The coil’s scalp location and trajectory were recorded using the BrainSight™ stereotactic system (Rogue Research, Montreal, QC, Canada). The same hotspot was used for all PA and AP pulse durations [21,25].

The TMS stimulator intensity (% of stimulator output) was defined separately for each combination of current direction and pulse duration, and for each muscle activation state. For the resting state session, the stimulator intensity was set to the stimulus intensity required to elicit an MEP of ~1 mV in the relaxed FDI using the maximum likelihood parameter-estimation (ML-PEST) adaptive parameter-estimation procedure [26]. For the contracted session, the stimulus intensity was set to the stimulus intensity required to elicit an MEP of ~1 mV while participants maintained a 10% MVC in the FDI muscle using the same ML-PEST method [26,27]. During the ML-PEST procedure, the study team monitored target contraction using continuous EMG recording and provided verbal feedback to help participants maintain it. Participants did not receive continuous visual feedback of their EMG signal.

Six separate stimulus intensities were independently defined within each session, corresponding to each combination of current direction and pulse duration (PA_30_, PA_70_, PA_120_, AP_30_, AP_70_ and AP_120_). Each stimulus intensity was determined immediately before the block of trials using that stimulation configuration. The intensity of the test stimulus was then held constant throughout the 156-trial block. In the event a 1 mV MEP could not be achieved with a given current direction-pulse duration combination, the session was conducted for all other current direction-pulse duration combinations.

During contracted data collection, target contraction was monitored by the study team using the continuous EMG recording, and verbal feedback was provided as needed. During offline analysis, background EMG activity in the baseline period before the TMS stimulus (−100 to 0 ms) was quantified using RMS amplitude. Trials with unusually high background muscle activity before stimulation were excluded from analysis. Excluded trials were defined as those with baseline root-mean-square amplitude exceeding the 75th percentile plus 3 times the interquartile range within a given condition (<1% of all trials).

### 2.4. Short-Latency Afferent Inhibition (SAI)

SAI was calculated as the MEP amplitude evoked by TMS stimuli conditioned by right wrist median nerve stimulation, expressed as a proportion of the unconditioned MEP amplitude. Electrical stimulation was delivered during conditioned trials using a DS7A constant-current, high-voltage stimulator (Digitimer, North America LLC, Fort Lauderdale, FL, USA). Stimulation was applied as a constant-current square-wave pulse with a duration of 0.2 ms, cathode-proximal. The peripheral electrical stimulus was set separately for each session to an intensity to achieve an abductor pollicis brevis M-wave of 0.2 mV. The same peripheral stimulus intensity was used for all TMS current direction-pulse duration configurations during a session.

For all TMS current-direction-pulse-duration configurations, the electrical stimulation of the median nerve preceded TMS stimulation by 10, 16, 18, 20, 22, 24, 26, 28, 30, 32, 34, or 36 ms [6,14,18,21]. Trial order was pseudorandomized. Six unique 13-trial sequences were generated, each containing one trial at each conditioned ISI and one unconditioned trial interspersed among the conditioned trials. ISIs were not presented in a fixed ascending or descending order. Each 13-trial sequence was then mirrored to create a 26-trial subblock, such that each conditioned ISI occurred twice and two unconditioned trials occurred within each subblock. The six 26-trial subblocks were concatenated to create a 156-trial block. Thus, each block contained 12 trials at each conditioned ISI and 12 unconditioned trials. The same pseudorandomized trial order was used for all combinations of muscle activation state, current direction, and pulse duration. The intertrial interval was sampled from a uniform distribution over the interval of 5 to 7 s, with a mean of 6 s. Each 26-trial subblock lasted approximately 2.5 min. Participants were given a break after every 26 trials and between each 156-trial block. Participants could also request a break at any point during the 26-trial subblock if needed.

### 2.5. Data Analyses

Analyses were conducted in R (version 4.4.3; R Core Team, Vienna, Austria) using the tidyverse, ggpubr, lme4, lmerTest, emmeans, and performance packages.

To assess the dynamics of SAI across the 10–36 ms ISI window, analyses were conducted at both the individual subject level using Gaussian curve fitting and at the group level using linear mixed-effects models. A Gaussian model was selected because prior work and the group-level SAI profiles suggest that SAI is typically expressed as a temporally constrained inhibitory response across the ISI range tested [3,6]. This approach provided a parsimonious way to summarize each participant’s SAI time course using three interpretable parameters: inhibition magnitude, peak timing, and temporal width.

For each participant and combination of State (Relaxed, Contracted), Current Direction (PA, AP) and Pulse Duration (30 µs, 70 µs and 120 µs), SAI was modelled as a function of ISI using a Gaussian function:

$$SAI=1-A\cdot exp\left(-\frac{{\left(ISI-\;\mu \right)}^{2}}{2\sigma^{2}}\right)$$

where A represents the magnitude of inhibition, μ the ISI of peak inhibition, and σ the width of the inhibition window (FWHM=2.355·σ). The dependent variable was the proportion of conditioned-to-unconditioned amplitude. Therefore, only the conditioned ISIs (10–36 ms) were included in the model fitting. Parameters were estimated using nonlinear least squares with bounded constraints (A: 0–1, μ: 18–28 ms, σ: 1.5–12 ms), with initial values derived from the physiologically relevant ISI window (18–28 ms). All Gaussian models fitted for each combination of current direction, pulse duration and muscle state across participants successfully converged. Fits were excluded from the cleaned analysis if the overall root-mean-squared error between the fit and the actual data, or the FWHM, exceeded the 1.5 × interquartile-range criterion for that condition. The root-mean-squared error criterion was applied to identify converged Gaussian models with unusually large residual error, indicating comparatively poor correspondence between the observed SAI values and the fitted curve. The FWHM criterion was applied to identify models that were reasonable fits but yielded an unusually wide or narrow inhibitory window. Of the 209 Gaussian curves, 18 were excluded. Fourteen curves were identified as outliers based on root-mean-squared error. Four curves were identified as outliers based on FWHM.

Effects of State, Current Direction and Pulse Duration on Gaussian parameters (A, μ and FWHM) were assessed using a linear mixed model with participant included as a random intercept. Statistical significance was evaluated using a Type III ANOVA with Satterthwaite approximations, based on all available data. Model performance was assessed using marginal and conditional R^2^ values and effect sizes for each term reported as partial eta-squared (η_p_^2^). Significant interactions were followed up using planned simple-effects contrasts based on estimated marginal means. Contrasts tested differences between current directions within each State × Pulse Duration condition, duration effects within each State × Current Direction condition, and State effects within each Current Direction × Pulse Duration condition. Contrast *p*-values were adjusted for multiple comparisons within each contrast family using the Benjamini–Hochberg false discovery correction, where appropriate.

An MEP of 1 mV was achieved for all PA pulse durations. However, for some participants, an MEP of 1 mV could not be achieved for AP stimulation before the maximal available stimulator output was reached. Therefore, SAI could not be assessed in all AP conditions for every participant. Data from three participants were missing for AP_30_ SAI at rest; however, SAI was assessed for these participants in all other PA and AP conditions. Data from one additional participant were missing for AP_30_ at rest, AP_70_ at rest, AP_120_ at rest, and AP_30_ during contraction.

To further contextualize the robustness and sensitivity of the primary findings, we conducted two additional sets of analyses. First, each primary linear mixed model was repeated in two sensitivity analyses: one excluding the participant with multiple missing AP conditions, and one excluding all participants with any missing condition-level data.

We conducted a simulation-based smallest detectable effect analysis for the primary model terms using custom Monte Carlo code in R (version 4.4.3). For each tested effect magnitude, 1000 datasets were simulated using the present study design and the variance structure estimated from the corresponding fitted model. The smallest condition difference or difference-in-differences detectable with 80% power at α = 0.05 was estimated. Observed effects were compared with the corresponding smallest detectable effects to contextualize the study’s sensitivity.

Additional methodological details on the missing-data sensitivity analyses and simulation-based smallest detectable effect analyses are provided in the Materials and Methods sections of the Supplementary Materials, Sections S1.1 and S1.2, respectively.

## 3. Results

**Figure 1:**
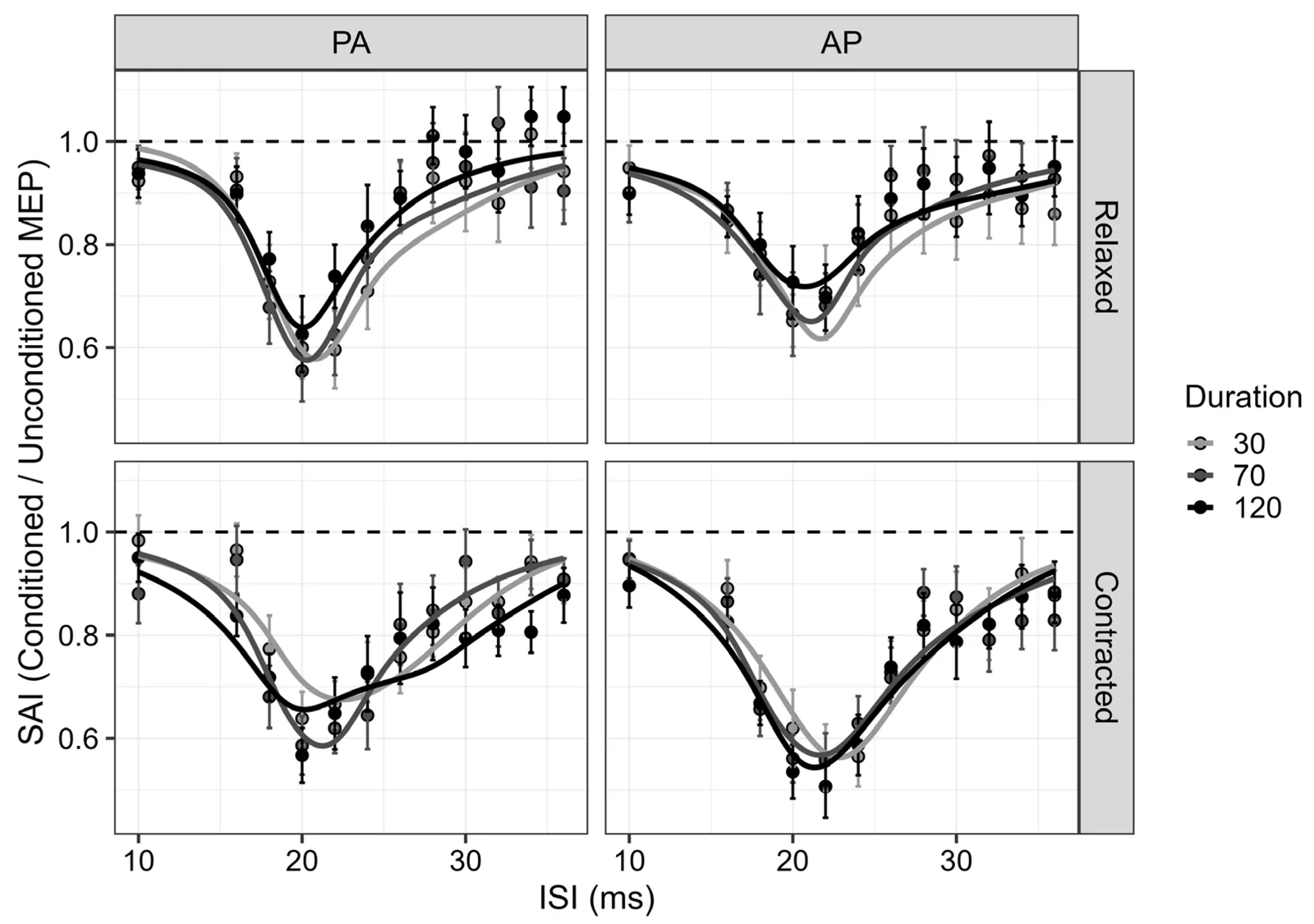
Mean SAI across ISIs for each combination of Current Direction (PA, AP), State (Relaxed, Contracted), and Pulse Duration (30, 70, 120). The lines represent the average of the individual fits. The dashed line represents the unconditioned MEP amplitude. Values close to 1 represent less inhibition. Error bars represent the standard error of the mean.

**Figure 2:**
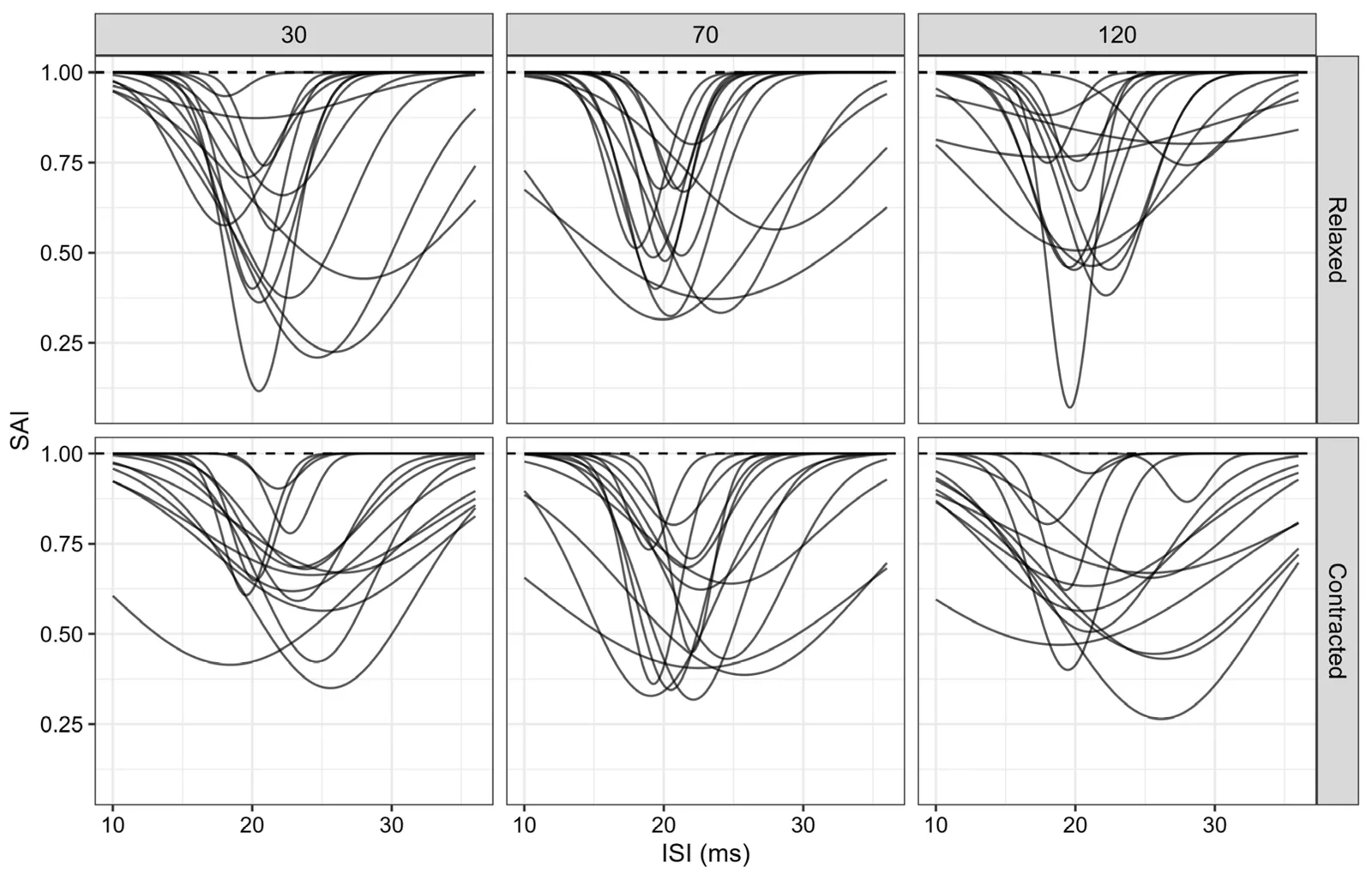
Individually fitted curves as a function of ISI for each participant for the PA Current Direction for each State (Relaxed, Contracted), and Pulse Duration (30, 70, 120). The dashed line represents the unconditioned MEP amplitude. Values closer to 1 represent less inhibition.

**Figure 3:**
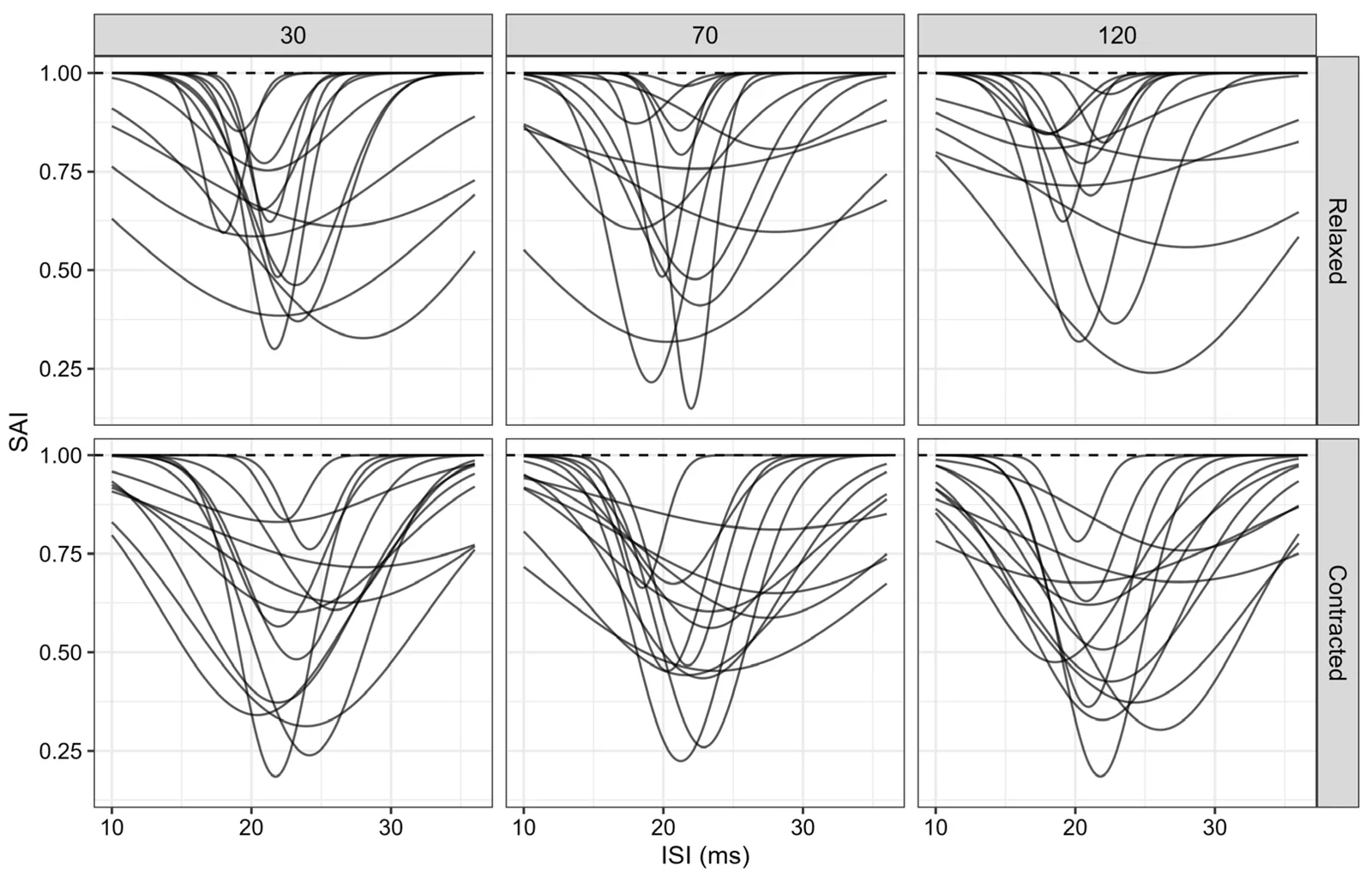
Individually fitted curves as a function of ISI for each participant for the AP Current Direction for each State (Relaxed, Contracted), and Pulse Duration (30, 70, 120). The dashed line represents the unconditioned MEP amplitude. Values closer to 1 represent less inhibition.

Figure 1 illustrates the grand-average SAI values across ISIs and the average of the individual fits for each current direction, pulse duration, and muscle state. Figure 2 and Figure 3 illustrate the individually fitted curves for each participant for the PA and AP current directions as a function of pulse duration and muscle state.

A linear mixed model with participant as a random factor on unconditioned MEP amplitude did not reveal any differences in unconditioned MEP amplitude across current direction, pulse duration, or muscle state (Supplementary Materials, Table S1). A similar linear mixed model was conducted on average baseline EMG activity during the pre-stimulus period before TMS onset (Supplementary Materials, Table S2). This analysis revealed a main effect of State, with greater baseline EMG activity in the contracted state compared with the relaxed state. Importantly, baseline EMG did not differ across current direction or pulse duration, and no interactions involving these factors were significant.

### 3.1. Peak Timing of Inhibition (μ)

**Figure 4:**
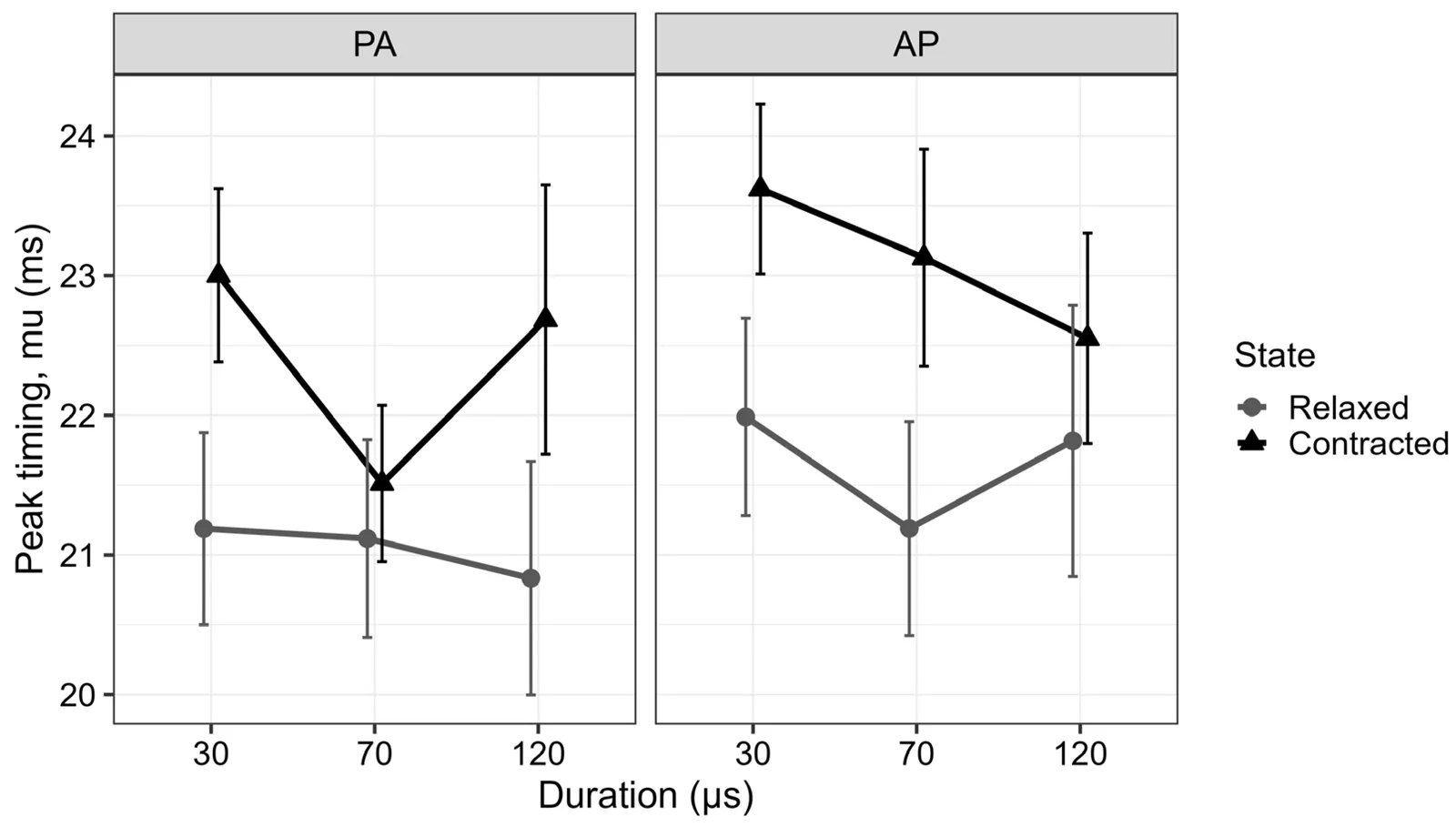
Estimated marginal means of peak timing of inhibition (μ) for the PA (**left panel**) and AP (**right panel**) Current Directions across muscle State (Relaxed, Contracted) and Pulse Duration (30 µs, 70 µs and 120 µs). Error bars denote standard error of the mean.

Figure 4 illustrates the peak timing of inhibition across different states, current directions, and pulse durations. The linear mixed model results for the State (Relaxed, Contracted), Current Direction (PA, AP), and Pulse Duration (30 µs, 70 µs, and 120 µs) on peak timing of inhibition are presented in Table 1.

There was a significant main effect of State. Peak inhibition occurred at later ISIs in the contracted compared to the relaxed state.

The estimated marginal means for the main effect of State are reported in the Supplementary Materials (Section S2.3). This file also provides the complete results for the missing-data sensitivity analyses and simulation-based smallest detectable effect analysis introduced in the Supplementary Materials, Sections S2.6.1 and S2.7.1, respectively.

### 3.2. Temporal Width of Inhibition (FWHM)

**Figure 5:**
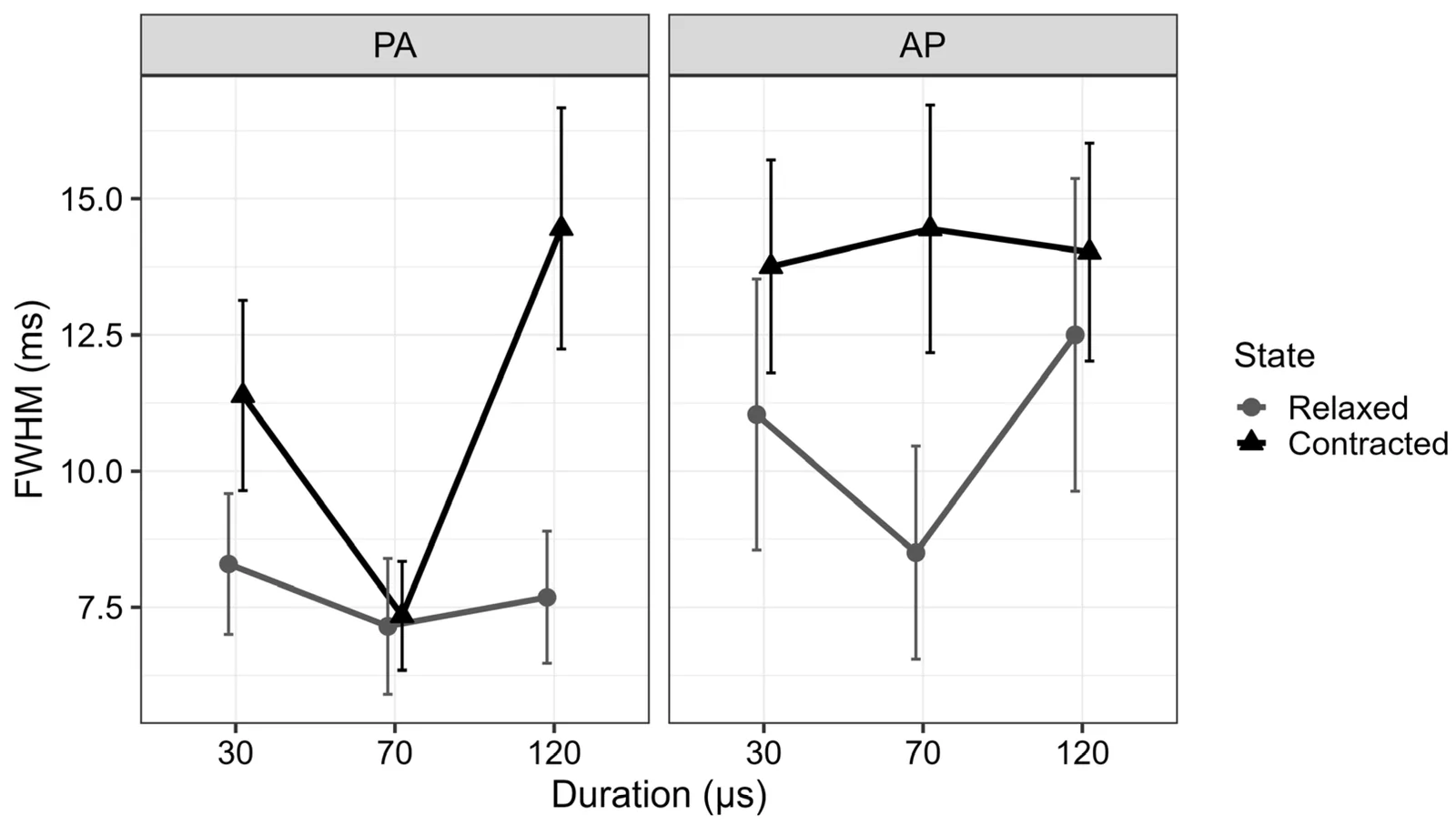
Estimated marginal means of the inhibition window (FWHM) for the PA (**left panel**) and AP (**right panel**) Current Directions across muscle State (Relaxed, Contracted) and Pulse Duration (30 µs, 70 µs and 120 µs). Error bars denote standard error of the mean.

Figure 5 illustrates the widths of the inhibitory windows across different states, current directions, and pulse durations. The linear mixed model results for the State (Relaxed, Contracted), Current Direction (PA, AP), and Pulse Duration (30 µs, 70 µs, and 120 µs) on FWHM are presented in Table 2.

Each of the main effects of State, Current Direction, and Pulse Duration was significant. The main effect of State was driven by a wider window of inhibition in the contracted compared to the relaxed state. The main effect of Current Direction was driven by a wider window of inhibition for AP than for PA. The main effect of Pulse Duration was driven by a narrower window of inhibition at 70 µs compared to both 30 and 120 µs (both contrasts, *p* = 0.02). The window was not different between 30 and 120 µs (*p* = 0.43).

The estimated marginal means for the significant main effects are reported in the Supplementary Materials (Section S2.4). This file also provides the complete results for the missing-data sensitivity analyses and simulation-based smallest detectable effect analysis introduced in the Supplementary Materials, Sections S2.6.2 and S2.7.2, respectively.

### 3.3. Magnitude of Inhibition (A)

**Figure 6:**
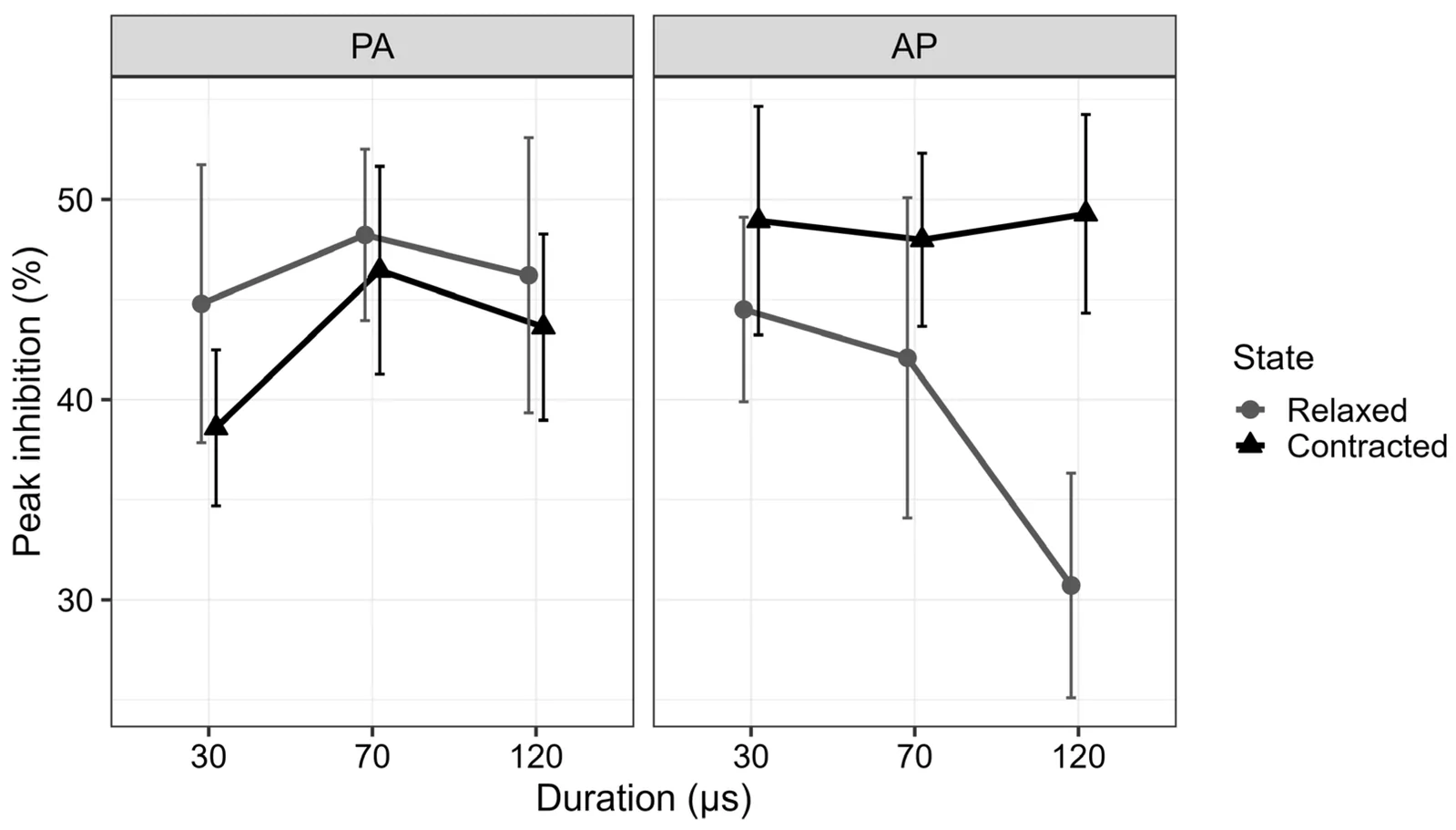
Estimated marginal means for the magnitude of inhibition (A) for the PA (**left panel**) and AP (**right panel**) across muscle State (Relaxed, Contracted) and Pulse Duration (30 µs, 70 µs and 120 µs). Peak inhibition is expressed as a percentage of the unconditioned MEP. Error bars denote standard error of the mean.

Figure 6 illustrates the magnitude of inhibition across different states, current directions, and pulse durations. The linear mixed model results for the State (Relaxed, Contracted), Current Direction (PA, AP), and Pulse Duration (30 µs, 70 µs, and 120 µs) on the magnitude of inhibition (A) are presented in Table 3.

There was a significant State × Current Direction interaction. The interaction was driven by an increase in the magnitude of inhibition from the relaxed to the contracted state for AP current regardless of pulse duration (*p* = 0.002). In contrast, there was no change in the magnitude of inhibition for PA current (*p* = 0.88).

The estimated marginal means for the State × Current Direction interaction are reported in the Supplementary Materials (Section S2.5). This file also provides the complete results for the missing-data sensitivity analyses and simulation-based smallest detectable effect analysis introduced in the Supplementary Materials, Sections S2.6.3 and S2.7.3, respectively.

## 4. Discussion

The present study investigated how SAI is shaped by muscle activation state, TMS current direction and pulse duration using cTMS. By sampling SAI across a broad range of ISIs and modelling the full time course of inhibition, we characterized changes in the timing, magnitude, and temporal width of early afferent inhibition in the motor cortex. The main findings were threefold. First, voluntary contraction shifted the timing of peak inhibition (μ) to later ISIs, independent of current direction and pulse duration. Second, the temporal width of inhibition (FWHM) increased during contraction and was broader for the AP current than for the PA current. Third, the magnitude of inhibition (A) was modulated by muscle state for AP stimulation, whereas PA SAI remained unchanged. Together, these findings indicate that SAI is not fixed across stimulation parameters or motor states and suggest that it may not represent a unitary phenomenon. Rather, the partially overlapping neural elements preferentially recruited by PA and AP stimulation may capture complementary aspects of how somatosensory afference shapes motor cortex excitability.

### 4.1. Muscle Activation State Shifts the Timing of Afferent Inhibition

A central finding was that voluntary contraction shifted the timing of peak inhibition toward later ISIs by approximately 1.3 ms. The shift occurred regardless of current direction or pulse duration. This consistency across stimulation configurations suggests a broad influence of muscle activation state on the temporal expression of SAI.

One physiologically plausible interpretation is that voluntary contraction changes the timing at which afferent input most strongly interacts with the neural elements contributing to the TMS-evoked response. Epidural recordings indicate that voluntary contraction alters TMS-evoked descending volleys. At 20% MVC, Di Lazzaro et al. reported only modest changes in the overall epidural volley recorded from the upper cervical spinal cord, with contraction-related facilitation being most evident in a later I-wave component [28]. Past work also suggests that SAI of PA-evoked responses is expressed predominantly through suppression of later I-wave components, rather than the earliest I1 component [6]. The magnitude of the contraction-related shift in peak SAI timing observed here (~1.3 ms) approximates the interval between successive indirect (I) waves elicited by TMS [29], raising the possibility that contraction altered the relative contributions of the neural elements through which afferent inhibition was expressed.

The current study did not record epidural volleys or single-motor-unit activity. Therefore, the shift in the timing of peak inhibition should not be interpreted as a direct mapping onto a specific I-wave. Rather, the shift provides evidence that the temporal dynamics of SAI are state-dependent, without establishing which TMS-recruited neural elements contribute to this effect [30,31]. This state dependence may be missed when SAI is assessed at a single ISI and may be particularly relevant during sustained, low-level contraction, where the motor system must maintain stable output while continuously integrating somatosensory feedback.

### 4.2. Temporal Width Differs Across Muscle States and Current Directions

In addition to shifting the timing of peak inhibition, voluntary contraction also increased the temporal width of the inhibitory window (FWHM). This finding indicates that SAI was expressed across a broader range of ISIs during active motor output. Such a widening of the inhibitory window may reflect a broader temporal range during which afferent input interacts with the neural elements that contribute to the TMS-evoked response during voluntary contraction.

A second key observation was that the temporal width of SAI was greater for AP stimulation than for PA stimulation, suggesting that the responses evoked by AP current were associated with a broader temporal expression of afferent inhibition. This broader temporal dispersion is consistent with previous evidence that AP SAI is sensitive to a range of experimental manipulations. For example, the cerebellar influence on SAI of AP-evoked responses appears to be stronger than that on PA-evoked responses [17,21]. Further, SAI of AP-evoked responses is more strongly modulated by attention load [17] and task demands [18], with cerebellar influences interacting with attention load [17]. In contrast, PA SAI appears to be more closely associated with functions such as preventing unwanted movements through surround inhibition [10], movement-related gating [9] and action sequencing [11]. The broader inhibitory window for AP SAI is therefore consistent with greater sensitivity of AP SAI to experimental and behavioral context.

We initially observed a weak trend toward significance in the interaction between induced current direction and pulse duration. Exploratory follow-up comparisons suggested that this pattern was primarily related to a wider AP SAI inhibitory window compared with PA SAI for the 70 µs pulse duration. However, sensitivity analyses indicated that this pattern was not robust after excluding participants with missing AP condition-level data. Therefore, the Current Direction × Pulse Duration pattern should be interpreted with caution as an exploratory observation rather than as evidence for a robust duration-specific dissociation. The basis of this non-robust pattern cannot be determined here, but it may be relevant given that the 70 µs duration approximates that of conventional TMS stimulators [14].

### 4.3. Selective Modulation of AP SAI by Muscle Activation State

The most notable dissociation involving current direction was observed for the magnitude of inhibition (A). The greater magnitude of AP SAI during voluntary contraction suggests that muscle activation influenced afferent inhibition differently for PA- and AP-evoked responses. Importantly, this does not imply that PA SAI was unaffected by voluntary contraction, because the timing of peak inhibition and the width of the inhibitory window were modulated for SAI of both PA- and AP-evoked responses. Rather, muscle activation state influenced different features of the SAI profile in different ways: timing and temporal width were broadly state-dependent, whereas inhibition magnitude was more strongly state-dependent for AP-evoked responses.

The selective magnitude effect extends the previously reported sensitivity of AP SAI to experimental and behavioral context to muscle activation state. In the present study, maintaining a specified level of contraction without continuous visual feedback likely increased the ongoing use of somatosensory information relative to rest. However, the design cannot determine whether the selective increase in AP SAI arose from changes in attentional demands, cerebellar influences, task requirements, or another source. The findings therefore indicate that the state dependence of SAI differed between PA- and AP-evoked responses, although the neural source of this difference cannot be determined from the present design.

### 4.4. Implications for Models of SAI

Together, the present findings support a more nuanced view of SAI as a temporally structured response whose expression depends on motor state and stimulation parameters. Rather than treating SAI as a single inhibitory value measured at a single ISI, the present results show that peak timing, inhibition magnitude, and temporal width provide complementary readouts of the SAI time course. The fact that these readouts were not uniformly affected by muscle state and current direction suggests they may capture distinct functional aspects of how somatosensory input modulates motor output. These features therefore distinguish state- and stimulation-dependent patterns of afferent inhibition that are not fully captured by single-ISI measures.

A useful interpretation is that PA and AP current directions differentially weight the contributions of partially overlapping neural elements to the TMS-evoked response. Within this framework, PA SAI may reflect a relatively temporally constrained form of afferent gating that is modulated by motor state, whereas AP SAI may be more sensitive to the broader sensorimotor context in which somatosensory afferent input is processed. This interpretation does not imply that each current direction isolates a discrete motor cortical circuit. The specific role of pulse duration remains less clear. In the present study, evidence for duration-specific effects was weak and was not robust in sensitivity analyses. It is possible that clearer pulse-duration dissociations may emerge under more complex behavioral contexts, but this remains to be tested.

This framework may help contextualize why SAI has been associated with sensory gating, movement preparation, attention, cerebellar modulation, and sensorimotor integration across studies. Variability between studies may partly reflect differences in the ISI, behavioral state and stimulation configuration, rather than inconsistency in a single fixed inhibitory process.

These considerations may also be relevant for future translational studies. SAI has been used to investigate abnormalities in cholinergic function, sensorimotor inhibition, and motor cortical excitability in clinical populations [2,32]. Although the present study was conducted in healthy adults and does not directly establish clinical utility, the findings suggest that clinical interpretations of SAI may depend on its measurement. In particular, single-ISI measurements at rest may not fully capture differences in the timing, magnitude, or temporal width of inhibition, nor the extent to which these features depend on motor state and stimulation parameters. Future studies in clinical populations may therefore benefit from profiling the SAI time course to determine whether disease-related changes are expressed primarily as altered inhibition magnitude, shifted peak timing, broadened or narrowed inhibitory windows, or altered state dependence.

### 4.5. Methodological Considerations and Limitations

Several methodological considerations should be noted. First, the inability to assess SAI in AP-evoked responses to short pulse durations at rest resulted in condition-specific missing data. This missingness was not random; it was concentrated under AP stimulation conditions, particularly AP_30_ at rest, consistent with past work [21]. Sensitivity analyses indicated that most primary findings were robust to the exclusion of participants with missing AP data, including the State effect on peak timing and the State × Current Direction interaction for peak inhibition magnitude. However, FWHM effects involving current direction were less stable across sensitivity analyses and should therefore be interpreted cautiously.

The loss of AP data may reflect a meaningful subgroup of participants who required higher AP stimulation intensities [33]. This is not unexpected for AP stimulation at short durations, as stimulus intensities are typically higher for this pulse configuration [18,21,33], particularly when using medium-inductance coils that resist rapid changes in current. The inability to assess SAI of AP-evoked responses at short pulse durations may have also reduced statistical power for effects involving the 30 µs pulse duration. More generally, although the repeated-measures design increased sensitivity, the modest sample size and condition-specific data loss, concentrated particularly in AP_30_, may have limited power to detect small interaction effects, especially those involving Current Direction and Pulse Duration. Weak or nonsignificant interactions should therefore not be interpreted as evidence that such effects are absent.

The smallest detectable effect analysis provided a similar interpretation. The study was well positioned to detect the observed State effect on peak timing, whereas the State × Current Direction effect for peak inhibition magnitude and the State and Current Direction effects for FWHM were closer to the detectable limits of the design. In contrast, pulse-duration-related FWHM effects, particularly the main effect of Duration and the Current Direction × Duration interaction, were smaller relative to the estimated detectable effects and were not robust across sensitivity analyses. These findings reinforce the exploratory status of the Current Direction × Pulse Duration pattern. Future work using low-inductance TMS coils, which offer less opposition to changes in current, may reduce missing data when assessing AP-evoked responses at short pulse durations.

Second, the Gaussian modelling approach assumes a unimodal inhibition profile that provides a useful summary of key parameters (μ, A and FWHM). However, this assumption may not fully capture the more complex, multipeaked dynamics observed in some individuals. We inspected individual fits, and there were occasional instances in which two peaks were evident. In these cases, one peak was typically dominant, and the model fits converged for every participant. To investigate the robustness of the Gaussian modelling approach, we repeated the analyses using linear mixed models, with SAI as the dependent variable and ISI as a categorical factor. We also conducted a generalized additive mixed model analysis, which does not impose a fixed symmetric curve. Both approaches produced results consistent with the Gaussian-based analyses. We therefore reported the Gaussian model-fitting results because they provided the simplest framework for capturing the effects of the independent variables on SAI magnitude, peak timing, and inhibitory-window width. Nevertheless, future work could more systematically explore alternative modelling approaches or non-parametric analyses to better characterize the functional significance of this individual variability.

Finally, although PA and AP stimulation are known to bias the recruitment of different neural elements, the present study did not directly measure which elements contributed to the TMS-evoked response or to its modulation by afferent input. The current-direction effects should therefore not be interpreted as direct evidence that specific circuits were involved.

## 5. Conclusions

In summary, the present study shows that characterizing a broader temporal profile of SAI provides insight into how somatosensory afferent input modulates TMS-evoked motor output across different motor states and stimulation parameters. By combining cTMS with modelling of the full SAI time course, this study moves beyond single-ISI measures. It demonstrates the value of estimating complementary features of the inhibitory profile, including peak magnitude, timing and temporal width. Voluntary contraction shifted the timing of peak inhibition and broadened the inhibitory window, whereas the magnitude of inhibition was most strongly modulated for AP-evoked responses. These findings indicate that the timing, magnitude, and temporal width of SAI may provide complementary information about early somatosensory influences on motor cortical excitability. More broadly, this methodological approach may provide a useful framework for future studies investigating sensorimotor function in both healthy and clinical populations.

## Figures and Tables

**Table 1:** The statistical results for the State × Current Direction × Pulse Duration linear mixed model on peak inhibition timing (μ). Degrees of freedom (df) are estimated using the Satterthwaite approximation (numerator, denominator). η_p_^2^ is the partial eta-squared for the specific effect. R^2^_M_ is the marginal R^2^, the variance explained by the fixed effects. R^2^_C_ is conditional R^2^, the variance explained by the full model, including the participant random factor. Effects highlighted in bold denote statistical significance.

Effect	df	F	*p*	η_p_^2^	R^2^_M_	R^2^_C_
**State**	**1,163**	**12.70**	**<0.001**	**0.072**	0.07	0.28
Current	1,164	1.80	0.18	0.011		
Duration	2,163	0.42	0.65	0.005		
State × Current	1,163	0.42	0.52	0.003		
State × Duration	2,163	0.13	0.88	0.002		
Current × Duration	2,164	0.12	0.89	0.001		
State × Current × Duration	2,163	0.43	0.65	0.005		

**Table 2:** The statistical results for the State × Current Direction × Pulse Duration linear mixed model for the temporal width of inhibition (FWHM). Degrees of freedom (df) are estimated using the Satterthwaite approximation (numerator, denominator). η_p_^2^ is the partial eta-squared for the specific effect. R^2^_M_ is the marginal R^2^, the variance explained by the fixed effects. R^2^_C_ is conditional R^2^, the variance explained by the full model, including the participant random factor. Effects highlighted in bold denote statistical significance.

Effect	df	F	*p*	η_p_^2^	R^2^_M_	R^2^_C_
**State**	**1,162**	**5.16**	**0.02**	**0.031**	0.12	0.27
**Current**	**1,163**	**7.46**	**0.007**	**0.044**		
**Duration**	**2,163**	**3.98**	**0.02**	**0.047**		
State × Current	1,163	0.01	0.93	<0.001		
State × Duration	2,163	0.22	0.80	0.003		
Current × Duration	2,163	2.86	0.06	0.034		
State × Current × Duration	2,163	0.79	0.45	0.010		

**Table 3:** The statistical results for the State × Current Direction × Pulse Duration linear mixed model for the magnitude of inhibition (A). Degrees of freedom (df) are estimated using the Satterthwaite approximation (numerator, denominator). η_p_^2^ is the partial eta-squared for the specific effect. R^2^_M_ is the marginal R^2^, the variance explained by the fixed effects. R^2^_C_ is conditional R^2^, the variance explained by the full model, including the participant random factor. Effects highlighted in bold denote statistical significance.

Effect	df	F	*p*	η_p_^2^	R^2^_M_	R^2^_C_
**State**	**1,162**	**4.31**	**0.04**	**0.026**	0.07	0.28
Current	1,162	0.28	0.60	0.002		
Duration	2,162	1.04	0.36	0.013		
**State × Current**	**1,162**	**5.19**	**0.02**	**0.031**		
State × Duration	2,162	2.06	0.13	0.025		
Current × Duration	2,162	1.08	0.34	0.013		
State × Current × Duration	2,163	0.13	0.88	0.002		

## Data Availability

The raw data supporting the conclusions of this article will be made available by the authors upon reasonable request.

## Supplementary Materials

The following supporting information can be downloaded at https://www.mdpi.com/article/10.3390/brainsci16080800/s1, Table S1: The statistical results for the State × Current Direction × Pulse Duration linear mixed model on the unconditioned motor evoked potential (MEP) amplitude; Table S2: The statistical results for the State × Current Direction × Pulse Duration linear mixed model on the unconditioned motor evoked potential (MEP) amplitude; Table S3: Sensitivity analyses for the primary linear mixed model terms for the timing of peak inhibition (*µ*). Table S4: Sensitivity analyses for the primary linear mixed model terms for the width of the inhibition window (FWHM); Table S5: Sensitivity analyses for the primary linear mixed model terms for the magnitude of inhibition (A); Table S6: Observed differences in peak inhibition timing compared with the smallest effects detectable with 80% power in the present design; Table S7: Observed differences in the width of the inhibitory window compared with the smallest differences detectable with 80% power in the present design; Table S8: Observed differences in peak inhibition magnitude compared with the smallest differences detectable with 80% power in the present design; File S1: Gaussian_Data_Mean_Clean.

## References

[1] Turco C.V., El-Sayes J., Savoie M.J., Fassett H.J., Locke M.B., Nelson A.J. (2018). Short- and long-latency afferent inhibition; Uses, mechanisms and influencing factors. Brain Stimul..

[2] Turco C.V., Toepp S.L., Foglia S.D., Dans P.W., Nelson A.J. (2021). Association of short- and long-latency afferent inhibition with human behavior. Clin. Neurophysiol..

[3] Tokimura H., Di Lazzaro V., Tokimura Y., Oliviero A., Profice P., Insola A., Mazzone P., Tonali P., Rothwell J.C. (2000). Short latency inhibition of human hand motor cortex by somatosensory input from the hand. J. Physiol..

[4] Chen R., Corwell B., Hallett M. (1999). Modulation of motor cortex excitability by median nerve and digit stimulation. Exp. Brain Res..

[5] Amassian V.E., Stewart M., Quirk G.J., Rosenthal J.L. (1987). Physiological basis of motor effects of a transient stimulus to cerebral cortex. Neurosurgery.

[6] Ni Z., Charab S., Gunraj C., Nelson A.J., Udupa K., Yeh I.J., Chen R. (2011). Transcranial magnetic stimulation in different current directions activates separate cortical circuits. J. Neurophysiol..

[7] Carrozzo C., Cannazza M., Fratini D., Fanella G., Cengiz B., Lazzaro V.D., Samusyte G., Tankisi H. (2026). The effects of caffeine on short-latency afferent inhibition measured with paired-pulse conventional and threshold-tracking TMS. Clin. Neurophysiol..

[8] Bailey A.Z., Asmussen M.J., Nelson A.J. (2016). Short-latency afferent inhibition determined by the sensory afferent volley. J. Neurophysiol..

[9] Asmussen M.J., Jacobs M.F., Lee K.G., Zapallow C.M., Nelson A.J. (2013). Short-latency afferent inhibition modulation during finger movement. PLoS ONE.

[10] Voller B., St Clair Gibson A., Dambrosia J., Pirio Richardson S., Lomarev M., Dang N., Hallett M. (2006). Short-latency afferent inhibition during selective finger movement. Exp. Brain Res..

[11] Suzuki L.Y., Meehan S.K. (2020). Attention focus modulates afferent input to motor cortex during skilled action. Hum. Mov. Sci..

[12] Asmussen M.J., Zapallow C.M., Jacobs M.F., Lee K.G.H., Tsang P., Nelson A.J. (2014). Modulation of Short-Latency Afferent Inhibition Depends on Digit and Task-Relevance. PLoS ONE.

[13] Dubbioso R., Raffin E., Karabanov A., Thielscher A., Siebner H.R. (2017). Centre-surround organization of fast sensorimotor integration in human motor hand area. NeuroImage.

[14] Lenizky M.W., Meehan S.K. (2024). The effects of verbal and spatial working memory on short- and long-latency sensorimotor circuits in the motor cortex. PLoS ONE.

[15] Alaydin H.C., Ataoglu E.E., Caglayan H.Z.B., Tokgoz N., Nazliel B., Cengiz B. (2021). Short-latency afferent inhibition remains intact without cortical somatosensory input: Evidence from a patient with isolated thalamic infarct. Brain Stimul..

[16] Oliviero A., Leon A.M., Holler I., Vila J.F., Siebner H.R., Della Marca G., Di Lazzaro V., Alvarez J.T. (2005). Reduced sensorimotor inhibition in the ipsilesional motor cortex in a patient with chronic stroke of the paramedian thalamus. Clin. Neurophysiol..

[17] Mirdamadi J.L., Meehan S.K. (2022). Specific sensorimotor interneuron circuits are sensitive to cerebellar-attention interactions. Front. Hum. Neurosci..

[18] Hayes K.D., Khan M.E.R., Graham K.R., Staines W.R., Meehan S.K. (2024). Persistent adaptations in sensorimotor interneuron circuits in the motor cortex with a history of sport-related concussion. Exp. Brain Res..

[19] Peterchev A.V., D’Ostilio K., Rothwell J.C., Murphy D.L. (2014). Controllable pulse parameter transcranial magnetic stimulator with enhanced circuit topology and pulse shaping. J. Neural Eng..

[20] D’Ostilio K., Goetz S.M., Hannah R., Ciocca M., Chieffo R., Chen J.A., Peterchev A.V., Rothwell J.C. (2016). Effect of coil orientation on strength-duration time constant and I-wave activation with controllable pulse parameter transcranial magnetic stimulation. Clin. Neurophysiol..

[21] Hannah R., Rothwell J.C. (2017). Pulse Duration as Well as Current Direction Determines the Specificity of Transcranial Magnetic Stimulation of Motor Cortex during Contraction. Brain Stimul..

[22] Rothkegel H., Sommer M., Paulus W., Lang N. (2010). Impact of pulse duration in single pulse TMS. Clin. Neurophysiol..

[23] MagVenture A/S (2010). MagPro Family User Guide 2010.

[24] Rossi S., Hallett M., Rossini P.M., Pascual-Leone A. (2011). Screening questionnaire before TMS: An update. Clin. Neurophysiol..

[25] Sakai K., Ugawa Y., Terao Y., Hanajima R., Furubayashi T., Kanazawa I. (1997). Preferential activation of different I waves by transcranial magnetic stimulation with a figure-of-eight-shaped coil. Exp. Brain Res..

[26] Silbert B.I., Patterson H.I., Pevcic D.D., Windnagel K.A., Thickbroom G.W. (2013). A comparison of relative-frequency and threshold-hunting methods to determine stimulus intensity in transcranial magnetic stimulation. Clin. Neurophysiol..

[27] Ah Sen C.B., Fassett H.J., El-Sayes J., Turco C.V., Hameer M.M., Nelson A.J. (2017). Active and resting motor threshold are efficiently obtained with adaptive threshold hunting. PLoS ONE.

[28] Di Lazzaro V., Restuccia D., Oliviero A., Profice P., Ferrara L., Insola A., Mazzone P., Tonali P., Rothwell J.C. (1998). Effects of voluntary contraction on descending volleys evoked by transcranial stimulation in conscious humans. J. Physiol..

[29] Di Lazzaro V., Oliviero A., Profice P., Saturno E., Pilato F., Insola A., Mazzone P., Tonali P., Rothwell J.C. (1998). Comparison of descending volleys evoked by transcranial magnetic and electric stimulation in conscious humans. Electroencephalogr. Clin. Neurophysiol. Mot. Control.

[30] Di Lazzaro V., Ziemann U. (2013). The contribution of transcranial magnetic stimulation in the functional evaluation of microcircuits in human motor cortex. Front. Neural Circuits.

[31] Ziemann U. (2020). I-waves in motor cortex revisited. Exp. Brain Res..

[32] Pascuzzi M., Naeini N., Dorich A., D’Angelo M., Kim J., Nankoo J.F., Desai N., Chen R. (2026). Versatility of Transcranial Magnetic Stimulation: A Review of Diagnostic and Therapeutic Applications. Brain Sci..

[33] Perrier M.L., Graham K.R., Vander Vaart J.E., Staines W.R., Meehan S.K. (2025). Sensitivity to Instruction Strategies in Motor Learning Is Predicted by Anterior-Posterior TMS Motor Thresholds. Brain Sci..

