# Supplementary material for: State-Dependent Temporal Dynamics of Short-Latency Afferent Inhibition Across Current Direction and Pulse Duration Using Controllable Pulse-Parameter TMS
Source: Brain Sci. 2026 Jul 29;16(8):800. doi: 10.3390/brainsci16080800 (PMC13511203; doi:10.3390/brainsci16080800)
Supplement: Supplementary file 1 [file brainsci-16-00800-s001.zip › brainsci-4323433-supplementary_proof_SKM.pdf]

S1. Supplementary Materials and Methods

S1.1 Missing-data sensitivity analyses

The primary linear mixed-effects models used all available Gaussian-fit data without imputation and included 18 participants. Because missing condition-level data were concentrated in the AP stimulation conditions, two sensitivity analyses were conducted to determine whether this pattern influenced the primary findings.

In the first sensitivity analysis, the participant missing AP_{30}, AP_{70}, and AP_{120} at rest and AP_{30} during contraction was excluded, resulting in a sample of 17 participants. In the second sensitivity analysis, this participant and the three additional participants missing AP_{30} at rest were excluded, resulting in a complete-case sample of 14 participants.

For each restricted dataset, the primary linear mixed-effects models for inhibition magnitude (A), peak timing (μ), and FWHM were repeated using the same model specification as the primary analyses. The effects of State, Current Direction and Pulse Duration were included as fixed effects. Participant was included as a random factor. Type III ANOVA tests with Satterthwaite-adjusted degrees of freedom were used to evaluate the fixed effects, and partial eta-squared (η_p^2) values were calculated for each model term. The statistical significance and effect-size estimates from the n = 17 and n = 14 analyses were compared with those from the primary all-available-data analysis to assess the robustness of the findings to the pattern of missing AP observations.

S1.2 Smallest Detectable Effect Analyses

A simulation-based smallest detectable effect analysis was conducted for each fixed-effect term in the primary linear mixed models of inhibition magnitude (A), peak timing (μ), and FWHM. The analysis was implemented using custom Monte Carlo code in R with the lme4, lmerTest, and tidyverse packages. Simulations were based on the cleaned Gaussian-fit datasets and retained the observed number of participants, repeated-measures structure, condition-level observations, and pattern of missing observations for each outcome. The participant random-intercept and residual standard deviations were extracted from the corresponding fitted model and used to generate normally distributed participant-level random intercepts and residual errors.

For each model term and tested effect magnitude, 1,000 datasets were generated using the observed grand mean, the imposed target effect, and the estimated random-intercept and residual variance components. The target effect was imposed in isolation, with the remaining fixed effects set to zero. The original linear mixed model, which included State, Current Direction, Pulse Duration, all interactions among these factors, and a participant-specific random intercept, was then refitted to each simulated dataset using restricted maximum likelihood. The target term was evaluated using a Type III F test with Satterthwaite-adjusted degrees of freedom. Power was estimated as the proportion of valid simulations in which the target term had $p < 0.05$.

Tested effect magnitudes ranged from 0 to 10 ms in 0.5 ms increments for peak timing, from 0 to 25 ms in 1 ms increments for FWHM, and from 0 to 0.50 in 0.025 increments for A, with A subsequently expressed as percentage points of peak inhibition. Main effects of State and Current Direction represented differences between their respective factor levels, whereas two-way interactions represented the corresponding differences-in-differences and the three-way interaction represented a difference-in-differences-in-differences. For effects involving Pulse Duration, a linear pattern was imposed by coding the 30, 70, and 120 μs conditions as −1, 0, and +1, respectively. Thus, the Pulse Duration effect represented the difference between 120 and 30 μs, with 70 μs positioned midway between them, and interactions involving Pulse Duration represented differences in this contrast. The smallest detectable effect was defined as the effect

associated with 80% power at α = 0.05 and was estimated by linear interpolation between the adjacent tested effect magnitudes immediately below and above 80% power. Observed effects were calculated from estimated marginal means using the same contrast definitions and compared with the corresponding smallest detectable effects.

S2. Supplementary Results

S2.1 Unconditioned MEP amplitude

Table S1. The statistical results for the State x Current Direction x Pulse Duration linear mixed model on the unconditioned motor evoked potential (MEP) amplitude. Degrees of freedom (df) were estimated using the Satterthwaite approximation (numerator, denominator). η_P^2 is the partial eta-squared for the specific effect. R^2_M is the marginal R^2, the variance explained by the fixed effects. R^2_C is conditional R^2, the variance explained by the full model, including the participant random factor.

Effect	df	F	p	η_P^2	R^2_M	R^2_C
State	1,181	0.37	0.54	0.002	0.03	0.14
Current	1,182	0.003	0.96	<0.001		
Duration	2,180	2.67	0.07	0.028		
State x Current	1,180	<0.001	0.98	<0.001		
State x Duration	2,180	0.52	0.60	0.006		
Current x Duration	2,180	0.09	0.92	<0.001		
State x Current x Duration	2,180	0.24	0.79	0.003		

S2.2 Pre-TMS stimulus EMG

Table S2. The statistical results for the State x Current Direction x Pulse Duration linear mixed model on the unconditioned motor evoked potential (MEP) amplitude. Degrees of freedom (df) were estimated using the Satterthwaite approximation (numerator, denominator). η_P^2 is the partial eta-squared for the specific effect. R^2_M is the marginal R^2, the variance explained by the fixed effects. R^2_C is conditional R^2, the variance explained by the full model, including the participant random factor. Effects highlighted in bold denote statistical significance.

Effect	df	F	p	η_P^2	R^2_M	R^2_C
**State**	**1,197**	**20.61**	**<0.001**	**0.095**	0.13	N/A
Current	1,197	0.76	0.38	0.003		
Duration	2,197	1.05	0.35	0.011		
State x Current	1,197	0.77	0.38	0.004		
State x Duration	2,197	1.05	0.35	0.011		
Current x Duration	2,197	0.99	0.37	0.010		
State x Current x Duration	2,197	0.96	0.38	0.010		

S2.3 Peak timing of inhibition (μ)

The main effect means for State were 21.8 ms [95% CI: 20.9 to 22.7] for the relaxed state and 23.1 ms [95% CI: 22.3 to 24.0] for the contracted state.

S2.4 Temporal width of inhibition (FWHM)

The means for the main effect of State were 8.45 ms [95% CI: 7.0 to 10.2] for the relaxed state and 11.1 ms [95% CI: 9.2 to 13.3] for the contracted state.

The main effect means for Current Direction were 8.7 ms [95% CI: 7.2 to 10.5] for PA current and 10.8 ms [95% CI: 8.9 to 13.0] for AP current.

The main effect means for Duration were 10.1 ms [95% CI: 8.24 to 12.4] for 30 μs, 8.1 ms [95% CI: 6.6 to 9.9] for 70 μs and 11.0 ms [95% CI: 9.0 to 13.5] for 120 μs.

S2.5 Magnitude of inhibition (A)

The State x Current Duration means for AP current were 0.38 [95% CI: 0.30 to 0.46] for the relaxed state and 0.47 [95% CI: 0.40 to 0.55] for the contracted state. For PA current, the means were 0.44 [95% CI: 0.36 to 0.52] for the relaxed state and 0.43 [95% CI: 0.36 to 0.52] for the contracted state.

S2.6 Missing-data sensitivity analyses

S2.6.1 Peak timing (μ)

Sensitivity analyses supported the robustness of the State effect on peak timing (Table S3). The effect remained significant after excluding the participant with multiple missing AP conditions and after excluding all participants with any missing condition-level data.

Table S3. Sensitivity analyses for the primary linear mixed model terms for the timing of peak inhibition (μ). η_P^2 indicates partial eta-squared. The original analyses are shown in the left columns. The n=17 columns show the p-values and effect sizes after excluding the participant missing AP_{30} rest, AP_{70} rest, AP_{120} rest and AP_{30} contraction. The n=14 columns show the p-values and effect sizes after additionally excluding the three participants with missing data for AP_{30} rest only. Effects highlighted in bold denote statistically significant effects from the original linear mixed model analyses reported in the main manuscript.

Effect	Original		n=17		n=14	
	p	η_P^2	p	η_P^2	p	η_P^2
**State**	**<0.001**	**0.072**	**0.007**	**0.045**	**0.001**	**0.084**
Current	0.18	0.011	0.22	0.010	0.10	0.021
Duration	0.65	0.005	0.47	0.010	0.37	0.015
State x Current	0.52	0.003	0.97	<0.001	0.94	<0.001
State x Duration	0.88	0.002	0.73	0.004	0.82	0.003
Current x Duration	0.89	0.001	0.91	0.001	0.84	0.003
State x Current x Duration	0.65	0.005	0.57	0.007	0.58	0.008

S2.6.2 Temporal width (FWHM)

Sensitivity analyses indicated that the main effects of State and Current Direction on FWHM were retained after excluding participants with missing AP data (Table S4). In contrast, effects involving Pulse Duration, including the main effect of Pulse Duration and the Current Direction × Pulse Duration pattern, were not robust across sensitivity analyses.

Table S4. Sensitivity analyses for the primary linear mixed model terms for the width of the inhibition window (FWHM). η_P^2 indicates partial eta-squared. The original analyses are shown in the left columns. The n=17 columns show the p-values and effect sizes after excluding the participant missing AP_{30} rest, AP_{70} rest, AP_{120} rest and AP_{30} contraction. The n=14 columns show the p-values and effect sizes after additionally excluding the three participants with missing data for AP_{30} rest only. Effects highlighted in bold denote statistically significant effects from the original linear mixed model analyses reported in the main manuscript.

Effect	All data		n=17		n=14	
	p	η_P^2	p	η_P^2	p	η_P^2
**State**	**0.02**	**0.031**	**0.07**	**0.021**	**0.004**	**0.063**
**Current**	**0.007**	**0.044**	**0.008**	**0.045**	**0.006**	**0.056**
**Duration**	**0.02**	**0.047**	**0.04**	**0.042**	**0.13**	**0.031**
State x Current	0.93	<0.001	0.71	0.001	0.99	<0.001
State x Duration	0.80	0.003	0.81	0.003	0.84	0.003
Current x Duration	0.06	0.034	0.13	0.026	0.72	0.005
State x Current x Duration	0.45	0.010	0.69	0.005	0.11	0.034

S2.6.3 Peak inhibition magnitude (A)

Sensitivity analyses supported the robustness of the State × Current Direction interaction for peak inhibition magnitude (Table S5). This interaction remained significant after excluding the participant with multiple missing AP conditions and after excluding all participants with any missing condition-level data.

Table S5. Sensitivity analyses for the primary linear mixed model terms for the magnitude of inhibition (A). η_P^2 indicates partial eta-squared. The original analyses are shown in the left columns. The n=17 columns show the p-values and effect sizes after excluding the participant missing AP_{30} rest, AP_{70} rest, AP_{120} rest and AP_{30} contraction. The n=14 columns show the p-values and effect sizes after additionally excluding the three participants with missing data for AP_{30} rest only. Effects highlighted in bold denote statistically significant effects from the original linear mixed model analyses reported in the main manuscript.

Effect	All data		n=17		n=14	
	p	η_P^2	p	η_P^2	p	η_P^2
**State**	**0.04**	**0.026**	**0.04**	**0.025**	**0.15**	**0.016**
Current	0.60	0.002	0.60	0.002	0.52	0.003
Duration	0.36	0.013	0.22	0.019	0.19	0.025
**State x Current**	**0.02**	**0.031**	**0.03**	**0.030**	**0.02**	**0.041**
State x Duration	0.13	0.025	0.20	0.021	0.20	0.024
Current x Duration	0.34	0.013	0.29	0.016	0.24	0.021
State x Current x Duration	0.88	0.002	0.76	0.003	0.58	0.008

S2.7 Smallest Detectable Effect Analyses

S2.7.1 Peak timing (μ)

The smallest detectable effect analysis indicated that the observed Muscle State difference was within the detectable range of the present design (Table S6).

Table S6. Observed differences in peak inhibition timing compared with the smallest effects detectable with 80% power in the present design. Values are reported as absolute differences in milliseconds. The smallest detectable difference represents the estimated condition difference, difference-in-differences, or higher-order interaction contrast required for each model term to be detected with approximately 80% power, given the present sample size and observed variance/covariance structure. For example, the smallest State × Current Direction × Pulse Duration interaction contrast detectable with approximately 80% power was estimated to be 6 ms, whereas the corresponding observed interaction contrast was 0.09 ms. Effects highlighted in bold denote statistically significant effects from the original linear mixed model analyses reported in the main manuscript.

Effect	Observed difference	Smallest Detectable Difference
**State**	**1.37**	**1.19**
Current	0.52	1.13
Duration	0.41	1.48
State x Current	0.50	2.20
State x Duration	0.24	2.96
Current x Duration	0.05	2.99
State x Current x Duration	0.09	6.00

S2.7.2 Temporal width (FWHM)

The smallest detectable effect analysis indicated that the observed differences for the main effects of Muscle State and Current Direction were within the detectable range of the present design (Table S7).

Table S7. Observed differences in the width of the inhibitory window compared with the smallest differences detectable with 80% power in the present design. Values are reported as absolute differences in milliseconds. The smallest detectable difference represents the estimated condition difference, difference-in-differences, or higher-order interaction contrast required for each model term to be detected with approximately 80% power, given the present sample size and observed variance/covariance structure. For example, the smallest detectable State × Current Direction × Pulse Duration interaction contrast was estimated to be 16.30 ms, whereas the corresponding observed interaction contrast was 6.55 ms. Effects highlighted in bold denote statistically significant effects from the original linear mixed model analyses reported in the main manuscript.

Effect	Observed difference	Smallest Detectable Difference
**State**	**2.40**	**2.95**
**Current**	**2.90**	**3.05**
**Duration**	**1.39**	**4.02**
State x Current	0.20	5.93
State x Duration	0.39	8.10
Current x Duration	2.67	8.15
State x Current x Duration	6.55	16.30

S2.7.3 Peak inhibition magnitude (A)

The smallest detectable effect analysis indicated that the observed interaction was close to the detectable range of the present design (Table S8).

Table S8. Observed differences in peak inhibition magnitude compared with the smallest differences detectable with 80% power in the present design. Values are reported as absolute percentage-point differences. The smallest detectable difference represents the estimated condition difference, difference-in-differences, or higher-order interaction contrast required for each model term to be detected with approximately 80% power, given the present sample size and observed variance/covariance structure. For example, the smallest detectable State × Current Direction × Pulse Duration interaction contrast was estimated to be 33.14 percentage points, whereas the corresponding observed interaction contrast was 4.86 percentage points. Effects highlighted in bold denote statistically significant effects from the original linear mixed model analyses reported in the main manuscript.

Effect	Observed difference	Smallest Detectable Difference
State	4.39	6.30
Current	1.12	6.22
Duration	2.13	8.30
**State x Current**	**9.66**	**11.61**
State x Duration	10.07	16.41
Current x Duration	6.63	16.41
State x Current x Duration	4.86	33.14
